# Human Brain Reacts to Transcranial Extraocular Light

**DOI:** 10.1371/journal.pone.0149525

**Published:** 2016-02-24

**Authors:** Lihua Sun, Jari Peräkylä, Anselmi Kovalainen, Keith H. Ogawa, Pekka J. Karhunen, Kaisa M. Hartikainen

**Affiliations:** 1 Behavioral Neurology Research Unit, Tampere University Hospital, Tampere, Finland; 2 John Magaddino Neuroscience Laboratory, Saint Mary’s College of California, Moraga, California, United States of America; 3 Department of Forensic Medicine, School of Medicine, Tampere University, Tampere University Hospital and Fimlab Laboratories, Tampere, Finland; 4 Department of Neuroscience and Rehabilitation, Tampere University Hospital, Tampere, Finland; Massachusetts General Hospital, UNITED STATES

## Abstract

Transcranial extraocular light affects the brains of birds and modulates their seasonal changes in physiology and behavior. However, whether the human brain is sensitive to extraocular light is unknown. To test whether extraocular light has any effect on human brain functioning, we measured brain electrophysiology of 18 young healthy subjects using event-related potentials while they performed a visual attention task embedded with emotional distractors. Extraocular light delivered via ear canals abolished normal emotional modulation of attention related brain responses. With no extraocular light delivered, emotional distractors reduced centro-parietal P300 amplitude compared to neutral distractors. This phenomenon disappeared with extraocular light delivery. Extraocular light delivered through the ear canals was shown to penetrate at the base of the scull of a cadaver. Thus, we have shown that extraocular light impacts human brain functioning calling for further research on the mechanisms of action of light on the human brain.

## Introduction

Ambient light guides behavior not only through the traditional visual pathway, but also by regulating numerous nonvisual physiological functions, including circadian, neuroendocrine, neurobehavioral responses and mood [1, 2]. While the main route for light to impact the brain is via photoreceptors on the retina, some birds are known to have deep brain photoreceptors in the hypothalamic and septal regions that react to transcranial extraocular light. These deep brain photoreceptors modulate seasonal changes in physiology and behavior of birds, such as seasonal breading [3, 4]. The effect of extraocular light on reproduction is most commonly studied on birds with eyes and pineal gland surgically removed to avoid ocular light interference. Long-term exposure to light is also needed for measurable changes in physiology [3–5]. Study of extraocular photosensitive proteins led to the discovery of deep brain photoreceptors in birds, e.g. Opsin 5, which detects blue and ultraviolet light and modulates seasonal breeding [3]. However, whether the human brain is sensitive to extraocular light is unknown and a similar approach of studying extraocular photosensitivity of birds is not applicable for humans.

In animals it has been shown that the skull is not completely impenetrable to light [4, 6] and that the measurable density of skull-penetrated light was found to affect neural metabolism. For example, transcranial bright light has been reported to enhance potassium-induced release of γ-aminobutyric acid (GABA) in cortical neurons of rats [7]. Penetration of light via the skull is also supported in a study showing that covering the head of blind ducks diminished photoperiodicity, where normal testicular growth in response to long daylight stimuli was abolished due to preventing the cranium from exposure to light [8]. Thus, although effect of the extraocular light is subtle it might be important in regulating physiological responses.

Human neuroimaging studies report that exposure to ocular blue light modulates brain activity, higher cognitive functions and emotional brain responses to auditory tasks in visually blind individuals [9–11]. This effect is believed to occur via a novel class of photosensitive retinal ganglion cells distinct from the rods and cones called intrinsically photosensitive retinal ganglion cells (ipRGCs) expressing the photopigment melanopsin that is maximally sensitive to short wavelength blue light (~480 nm) [12]. While amphibians, fish, reptiles and birds have been reported to possess deep brain photoreceptors that mediate behavior [13, 14], it is thought mammals lack a similar photosensitive receptor and the evidence for a homologous receptor in mammals remains circumstantial [15–17]. Further, it is neither known whether the comparatively thick human skull bone is penetrable by visible light.

In order to study whether there is any effect of extraocular light on human brain function, we investigated the effect of extraocular light on brain’s electrophysiology using event-related potentials (ERPs) and on behavior. ERPs are well suited for studying potential effects of extraocular light on brain physiology with vastly studied components such as the P300 sensitive to attentional and cognitive processes as well as biological and environmental factors [18]. With centro-parietal distribution of the classical P300 or P300b we chose P300 amplitude in the centro-parietal region of interest as a measure for the possible effect of extraocular light. In the present study, transcranial extraocular light was delivered via the ear canals and subject EEG was recorded while they performed a computer based Go/NoGo visual attention task embedded with emotional distractors, i.e. the Executive—Reaction Time (RT) test. Combined with EEG the Executive—RT test allows the study of subtle alterations in attention related brain responses and how they are modulated by emotional stimuli [19–21]. We investigated whether the transcranial extraocular light had any effect on centro-parietal P300 amplitude evoked in the Executive-RT test. Hypothesizing that extraocular light has an effect on brain physiology we expected this effect would be reflected in P300 amplitude and/or performance of the task. We further investigated whether the ear-canal-delivered light could penetrate the base of a skull in a human cadaver.

## Methods

### Subjects

Eighteen young healthy subjects (mean age = 25 y, sd = 6y, 3 male and 15 female) provided their written consent and voluntarily participated in the study according to the guidelines set forth in the Declaration of Helsinki governing the treatment of human subjects. The study was approved by the Regional Ethical Committee of Tampere University Hospital, Tampere, Finland and the permission number is R12237.

### Extraocular light delivery

**Fig 1:**
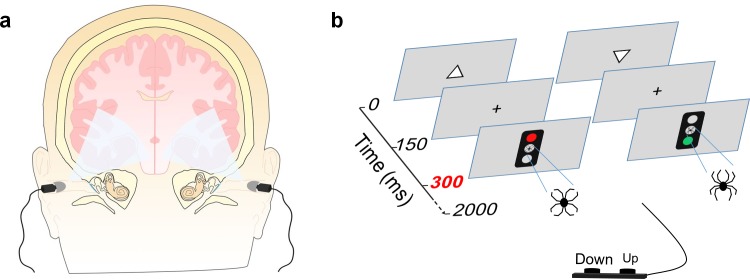
Experimental design. (A) Theoretical penetration of light via ear canals. (B) Schematic presentation of the Executive-RT test. In case of a Go trial subjects were required to report the orientation of a previously presented triangle pointing either up or down with a corresponding button press. In NoGo trials subjects were required to withhold from responding. The Go/NoGo signal was a green or a red traffic light embedded with an emotional (i.e. a line-drawing of a spider) or neutral distractor.

Extraocular light was delivered using a commercial Bright Light Ear Headset (NPT1100, Valkee Oy, Oulu, Finland; Fig 1A). UV-free and blue-enriched LED light with maximum of 3.5 Lumens was presented via both ear canals. The light has a photon density of 1.13 × 10^16^ photons ∙ cm^-2^ ∙ s^-1^ with a peak in the blue region around 448 nm. Detailed information of the LED light can be found in the previous study by Jurvelin et al. [22].

### The Behavioral Test

The behavioral test was conducted in a sound-attenuated room under soft white ceiling light. During the behavioral test, i.e. the Executive-RT test (Fig 1B), subjects sat one meter in front of a 21” computer screen. Presentation software (Neurobehavioral System, Inc.) was used to present the visual stimuli and collect the behavioral data. The first visual stimulus is a triangle (pointing either up or down) lasting 150 ms in the center of the screen. Thereafter, there was a 150 ms delay with a fixation cross in the center of the screen before onset of the Go/NoGo signal, i.e. the traffic light. The traffic light was presented for 150 ms leaving approximately 1550 ms for response before the next trial. Each trial lasts for 2000 milliseconds, with each block consisting of 64 trials and a total of 16 blocks. The response rules was switched between each block, i.e. if a green traffic light was the Go signal in the first block, then a red traffic light was the Go signal for the following block. Presented in the middle of the traffic light was a distractor, a schematic drawing in the shape of either a spider (emotional, threatening) or a flower (neutral, non-threatening) [23, 24]. The order of the Go/NoGo signals and the emotional/neutral distractors were randomized.

Prior to testing, the Bright Light headset ear plugs were placed into the ear canals of the subject. When the headset was ON, extraocular light was delivered and when it was OFF, no light was delivered. Light was either ON or OFF for approximately six minutes, thereby allowing subjects to finish two blocks of behavioral tests; thereafter the light status alternated. Eight blocks were completed with ON status and the remaining half of the test completed with OFF status. Subjects did not know whether the ear-canal light delivery was on or off.

### EEG recordings and data processing

Continuous electroencephalography (EEG) was recorded using a 64-channel actiCAP Ag/AgCl electrodes (Gilching, Germany) and digitized at 500Hz. Impedance for all electrodes was kept below 5 kΩ. Common reference was used during recording and in offline EEG data analysis. The offline EEG signal was processed using BrianVision Analyzer 2 (Brain Products, Gilching, Germany) software for event-related potential (ERPs) study. The signal was down sampled to 250Hz. Ocular movement correction was performed using “ICA ocular correction” function of Brain Analyzer 2, where EEG was decomposed into independent components using extended Informax algorithm and components (typically one or two) corresponding to artifact due to ocular movement were rejected. Following this, a band-pass filter (0.01–30 Hz) was applied. After filtering the EEG was then segmented into 2200 ms segments with 200 ms baseline before onset of the trial. The segments were baseline-corrected and processed for further artifact rejection, where segments with EEG amplitude higher than ±70 μV were rejected. ERPs were yielded by averaging the remaining segments for each condition. There were eight condition combinations composed of two types of emotional distractors, two extraocular light statuses and two response types.

ERP amplitude of the centro-parietal brain region of interest (covering electrodes C1, Cz, C2, CP1, CPz, CP2, P1, Pz and P2) was analyzed. In the ERP time window analysis, average amplitude of each 100-ms ERP window was exported for analysis. In P300 peak analysis, P300 peak was detected as the biggest positive peak between 300 ms and 550 ms after onset of the traffic light (i.e. the Go/NoGo signal), corresponding to 600–850 ms on the ERP real-time window. Detected P300 peaks were visually inspected. The P300 amplitude was an average amplitude of 20 ms around the detected peak.

### Statistical methods

Repeated-measure-analysis of variance (ANOVA) was used for analyzing ERPs and for reaction times in the behavioral test. Analysis of ERPs, including both analysis of both P300 amplitude and ERP time window analysis, was done using Extraocular light (ON vs. OFF), Emotion (neutral vs. emotional) and Response type (Go vs. NoGo) as factors.

In the reaction time analysis, we only involved trials of correct button press with reaction time longer than 150 ms. Analysis of reaction times was done using Extraocular light and Emotion as factors.

Logistic regression analysis was used for analyzing behavioral error types. Two categories of trials (Go/NoGo) generated three types of errors: incorrect button press (i.e. incorrect report of triangle orientation in Go trial), miss (i.e. no button press in Go trial) and commission errors (i.e. any button press in NoGo trial). Separate binary logistic regression models were generated for each error type. For each error type, trials were dichotomized into either “error” (e.g. incorrect button press in Go trials) or “other” (other outcome of Go trails, i.e. miss or correct response). Subject, Extraocular light, Emotion and interaction between Emotion and Extraocular light were used as predictors.

To account for multiple comparisons, the significance criteria was Bonferroni-adjusted to 0.006. All statistical analysis was performed in using R (version 3.1.3) with ez-package (version 4.2–2) [25].

### Skull penetrability to light

We included an autopsy case with the base of skull photographed in order to determine the penetrability of light. This case belongs to the Tampere Sudden Death Study (TSDS), where people died out-of-hospital and underwent medicolegal autopsy at the Department of Forensic Medicine, School of Medicine, University of Tampere. The study protocol was approved by the Regional Ethical Committee of Tampere University Hospital with permission number R09097. In Finland when a study has ethical committee approval and an autopsy is routinely done as is the case with sudden death, no next of kin consent and no previous permission from the diseased subject or their relatives is required, similarly as previous studies [26–29].

## Results

### Analysis of centro-parietal P300

**Fig 2:**
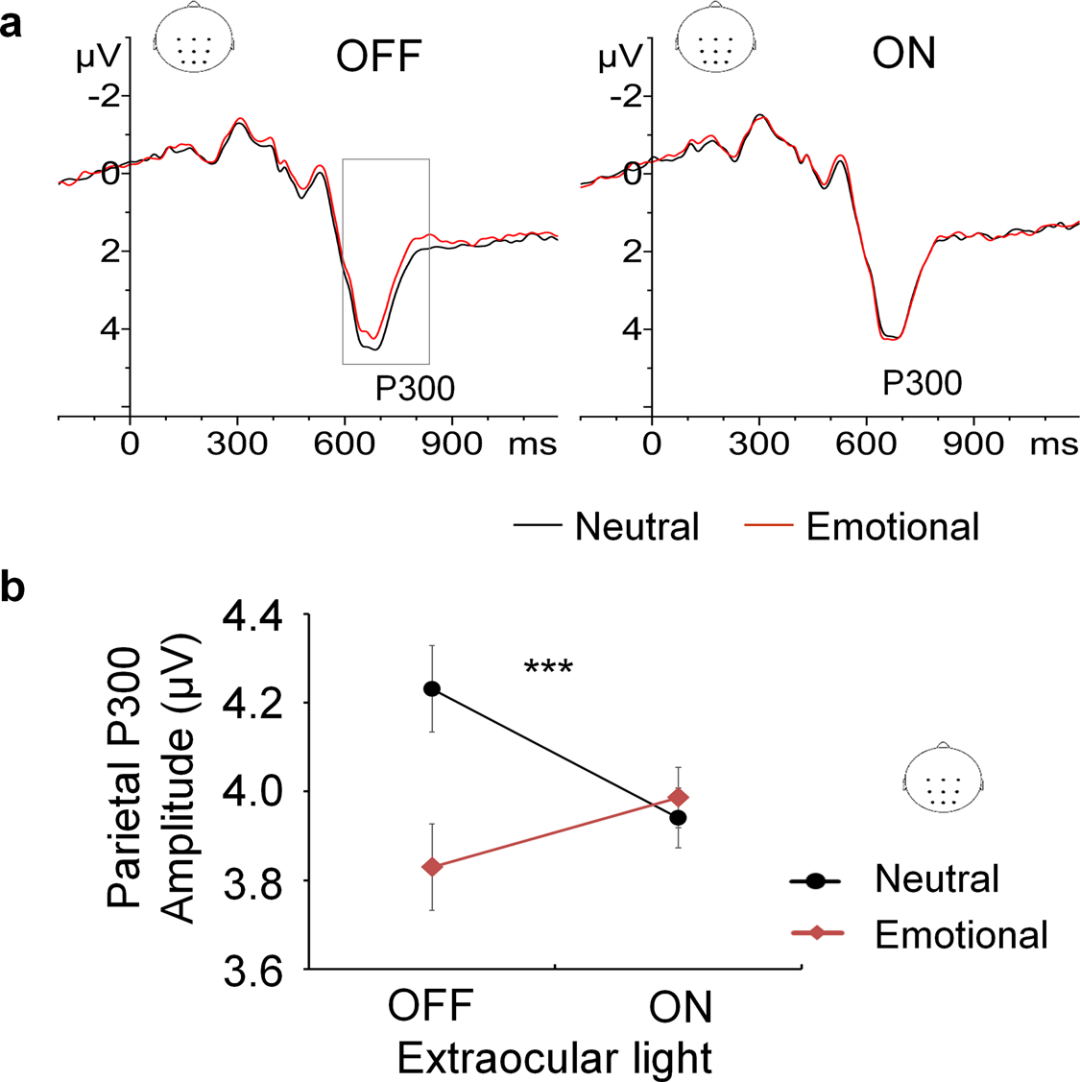
The effect of extraocular light on emotional modulation of P300 amplitude. (A) Grand-average ERP of the centro-parietal region. When extraocular light was OFF, emotional distractors diminished P300 amplitude compared to neutral distractors. When extraocular light was ON the valence of the distractor had no effect on the P300 amplitude. (B) Extraocular light abolished the normal emotional modulation of centro-parietal P300 amplitude. This effect was highly significant (p = .0001). Error bars: Fisher's least significant difference.

Analysis of centro-parietal P300 amplitude revealed a significant interaction between Extraocular light and Emotion, F (1, 17) = 25.48, p = .0001. Post hoc analysis revealed the main effect of Emotion existed only in situations with no extraocular light delivered, F (1, 17) = 10.83, p = .004. In contrast, when extraocular light was delivered, no effect of Emotion on P300 amplitude was found, F (1, 17) = 2.48, p = .13 (Fig 2).

### ERP time window analysis

**Fig 3:**
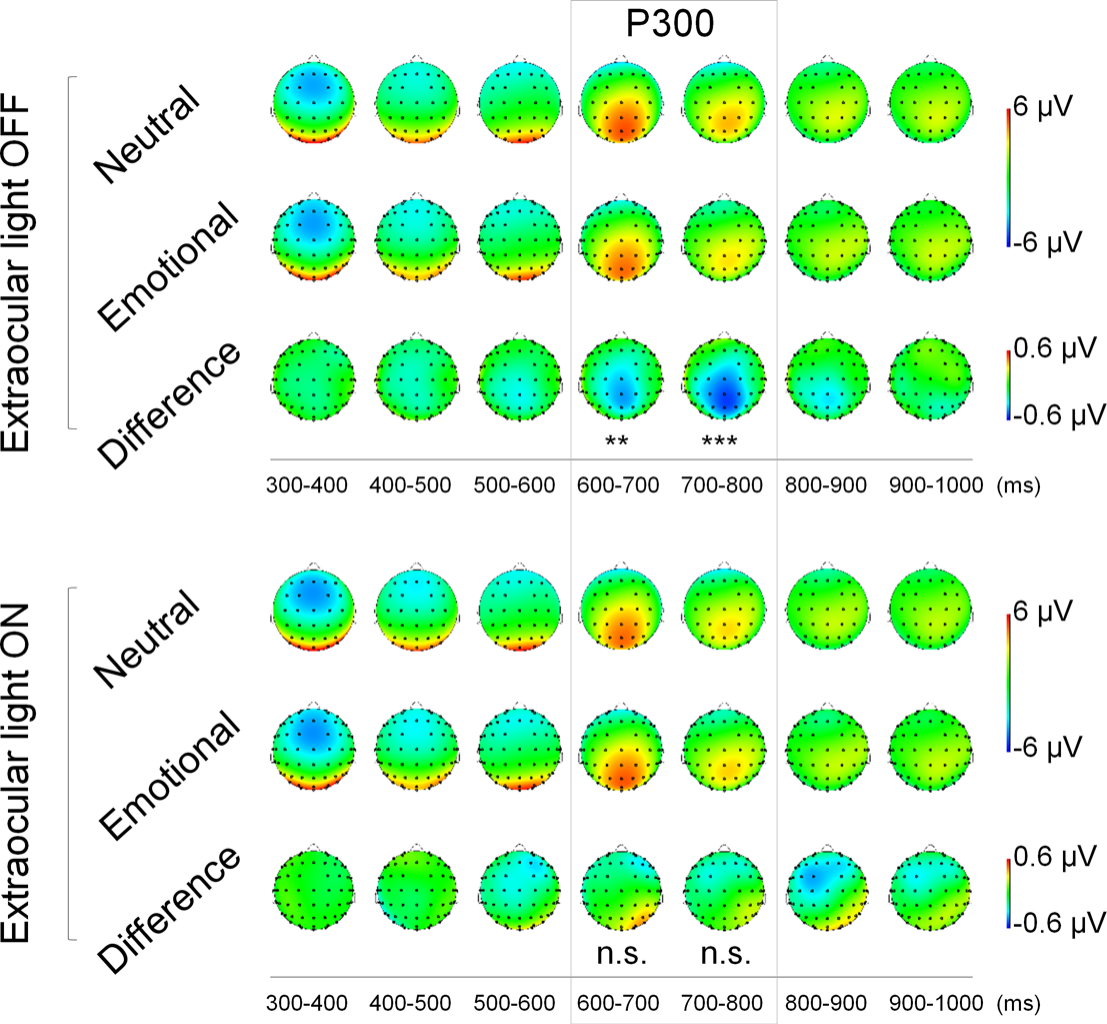
Extraocular light abolished the normal emotional modulation of attention related ERPs at 600–800 ms (corresponding to the 300–500 ms after Go/NoGo signal). When extraocular light was OFF (upper panel), the valence of the distractor had an effect on the ERP waveforms with Difference waveform (Emotional-Neutral) leading to centro-parietal negativity at 600–800ms. In contrast when extraocular light was ON (lower panel) the valence of the distractor did not have a significant effect on ERPs. The main effect of Emotion is marked; n.s. = no significance.

ERP time window analysis with 100-ms windows was also performed for the brain centro-parietal region (see also S1 Text and S1 Table). Analysis of ERP time windows revealed an interaction effect between Emotion and Extraocular light within two consecutive time windows 600–700 ms (F (1, 17) = 13.22, p = .002) and 700–800 ms (F (1, 17) = 12.86, p = .002). Post hoc analysis resulted in a main effect of Emotion when Extraocular light was OFF, but not when it was ON (Fig 3). These two consecutive time windows correspond to the latency of P300.

### Behavioral analysis

In the behavioral data analysis, no effects were found due to delivery of extraocular light (S2 Text and S2 Table).

### Transcranial light penetration via human ear canals

**Fig 4:**
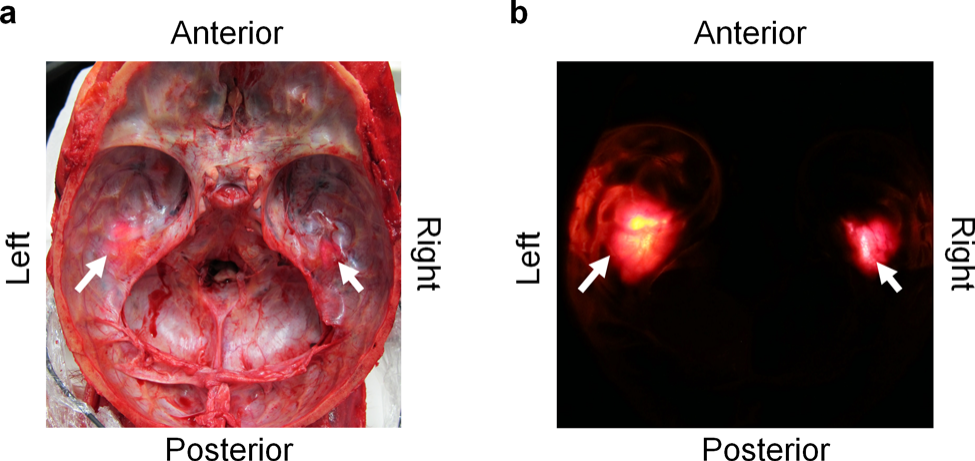
Light is able to penetrate human skull via ear canals. (A) Light penetration through the ear canals at the base of the scull on a cadaver after inserting the Bright Light Ear Headset into both ear canals under normal surgical lights in the autopsy room and (B) same skull base after turning the surgical lights off and darkening the room.

Possible penetration of light through the ear canals was investigated on a human cadaver after the brain was removed upon autopsy. Light penetration of the skull was visible when viewed both in lighted (Fig 4A) and dark (Fig 4B) conditions. Light was able to reach intracranial space through the ear canals and was visible at the base of the skull under the temporal lobes.

## Discussion

The present study demonstrates that extraocular light affects human brain functioning. Extraocular light modulated attention-related brain responses, specifically related to emotion-attention interaction. This light abolished emotional modulation of centro-parietal P300 brain response suggesting the extraocular photosensitivity of the brain. The light was able to penetrate the base of the skull and reach the brain's temporal lobes, as demonstrated in a human cadaver (Fig 4). Uncovering the subtle effect of extraocular light leads to new insight of human brain functioning.

We confirmed in our investigation of a human cadaver skull that light is capable of penetrating the human skull via ear canals and reach the temporal lobe of the brain. Because the brain is floating in cerebrospinal fluid, transmitted light might be widely dispersed, thus illuminating the basal surface of temporal lobe. The mechanism by which extraocular light may affect human brain functioning is unclear.

There might be several mechanisms of action of light on brain function depending on species, cell type and the physical properties of light. Deep brain photosensitive molecules OPN3 and OPN5 have been shown in mice and birds acting as signal transmitters for light [3, 30, 31]. Visible light has been reported to enhance potassium induced release of the neurotransmitter GABA of cortical neurons of rats [7]. On the other hand, near-infrared (NIR) light, found to increase adenosine triphosphate production in mitochondria, modulate reactive oxygen species and induce cellular transcription factors [32, 33]. NIR also penetrates human skull [34] and is used to study human brain hemodynamics (NIR spectroscopy) [35]. Furthermore, transcranial NIR has been studied as a potential therapy to treat mild traumatic brain injuries [36, 37].

Event-related potentials provide online information of brain functioning even with no behavioral changes [38–40]. In our study, ERP analysis revealed that extraocular light abolished the modulatory effect of emotion typically found on P300 amplitude [41–44]. This finding is consistent with previous EEG and fMRI studies showing changes in brain activity with ocular blue light exposure of less than a minute in visually blind subjects performing an auditory task [9]. Meanwhile, exposure to extraocular light delivered via the auditory canal did not alter performance on an Executive-RT task engaging several executive functions with threat-related and emotionally neutral distractors. The lack of behavioral signs might be due to extraocular light effects being subtle and the insensitive nature of behavioral measures. This is also in line with a previous study by Bromundt et al using light delivered via the auditory canal that found no evidence of performance improvement on a 10-minute psychomotor vigilance task [16]. Nevertheless, brain neuroimaging findings of these subtle effects, both due to extraocular and ocular light exposure, are likely to be demonstrated only when subjects are actively engaged in a cognitive task as was the case in our study and the previous ones [9, 10].

Beyond the apparent consistency with previous findings [9, 10, 16], uncovering the extraocular pathway of light in the human brain is revolutionary. With extraocular bright light delivery via both ear canals, centro-parietal P300 responds differently toward emotional distractors, indicating that the human brain reacts to extraocular light. The centro-parietal P300 has been associated with attentional resource allocation [45], with emotional stimuli able to capture attentional resources [19, 23, 46] and modulate centro-parietal P300 amplitude [41–43]. The emotional modulation of centro-parietal P300 amplitude due to emotional distractors disappeared during extra-ocular light delivery. Thus, extraocular light modulated emotion-attention interaction.

The current study has demonstrated that extraocular light has immediate effects on brain potentials of healthy subjects. Uncovering that brain functions may be modulated by extraocular bright light has broad implications for future research on brain physiology. Furthermore, our findings might also promote investigation on potential clinical applications. Whether chronic bright light delivery via the ear canals bears clinically applicable benefits is beyond the scope of this study. Transcranial bright light treatment has been previously reported by Jurvelin et al to relieve depressive symptoms associated with seasonal affective disorder [47]. While the study by Jurvelin et al lacked an adequate placebo control group and there was no dosage effect, the results suggested that transcranial light might impact mood. The current findings showing altered emotion-attention interaction due to transcranial light is consistent with potential effect of bright light on mood.

In conclusion, we have found that extraocular light impacts human brain physiology. Whether similar photosensitive brain receptors exist in the human brain as in birds is still unclear. The results from this study call for future research on the mechanism of action of light on the human brain. Demonstrating how extraocular light influences emotional reactivity might provide additional insights regarding how light directly affects mood [2]. The subtle effect of extraocular light might be critical for healthy human brain functions and disease. Therefore, the results from this study have potential widespread impact on understanding the effect of light on the healthy brain as well as its potential involvement in brain disorders.

## Supporting Information

S1 Table List of p values for the analysis of ERP time windows. (DOCX)

S2 Table Logistic regression analysis of error types did not reveal significant predictors. (DOCX)

S1 Text Analysis of Event-related potential data. (DOCX)

S2 Text Analysis of reaction times. (DOCX)

## References

[1] Hanifin JP, Brainard GC. Photoreception for circadian, neuroendocrine, and neurobehavioral regulation. Journal of physiological anthropology. 2007;26(2):87–94. . 17435349 10.2114/jpa2.26.87

[2] LeGates TA, Fernandez DC, Hattar S. Light as a central modulator of circadian rhythms, sleep and affect. Nature reviews Neuroscience. 2014;15(7):443–54. 10.1038/nrn3743 24917305 PMC4254760

[3] Nakane Y, Ikegami K, Ono H, Yamamoto N, Yoshida S, Hirunagi K, et al A mammalian neural tissue opsin (Opsin 5) is a deep brain photoreceptor in birds. Proceedings of the National Academy of Sciences of the United States of America. 2010;107(34):15264–8. 10.1073/pnas.1006393107 20679218 PMC2930557

[4] Foster RG, Follett BK, Lythgoe JN. Rhodopsin-like sensitivity of extra-retinal photoreceptors mediating the photoperiodic response in quail. Nature. 1985;313(5997):50–2. . 3965970 10.1038/313050a0

[5] Siopes TD, Wilson WO. Extraocular modification of photoreception in intact and pinealectomized coturnix. Poultry science. 1974;53(6):2035–41. . 4462102 10.3382/ps.0532035

[6] Van Brunt EE, Shepherd MD, Wall JR, Ganong WF, Clegg MT, editors. Penetration of light into the brain of mammals New York: Annals of the New York Academy of Sciences.

[7] Wade PD, Taylor J, Siekevitz P. Mammalian cerebral cortical tissue responds to low-intensity visible light. Proceedings of the National Academy of Sciences of the United States of America. 1988;85(23):9322–6. 3194426 10.1073/pnas.85.23.9322 PMC282731

[8] Benoit J. Le role des yeux dans l'action stimulante de la lumiere sure le developpement testiulaire chez le canard. CR Soc Bio (Paris). 1935;118:669–71.

[9] Vandewalle G, Collignon O, Hull JT, Daneault V, Albouy G, Lepore F, et al Blue light stimulates cognitive brain activity in visually blind individuals. Journal of cognitive neuroscience. 2013;25(12):2072–85. 10.1162/jocn_a_00450 23859643 PMC4497579

[10] Vandewalle G, Gais S, Schabus M, Balteau E, Carrier J, Darsaud A, et al Wavelength-dependent modulation of brain responses to a working memory task by daytime light exposure. Cerebral cortex. 2007;17(12):2788–95. 10.1093/cercor/bhm007 . 17404390

[11] Vandewalle G, Schwartz S, Grandjean D, Wuillaume C, Balteau E, Degueldre C, et al Spectral quality of light modulates emotional brain responses in humans. Proceedings of the National Academy of Sciences of the United States of America. 2010;107(45):19549–54. 10.1073/pnas.1010180107 20974959 PMC2984196

[12] Schmidt TM, Chen SK, Hattar S. Intrinsically photosensitive retinal ganglion cells: many subtypes, diverse functions. Trends Neurosci. 2011;34(11):572–80. 10.1016/j.tins.2011.07.001 21816493 PMC3200463

[13] Peirson SN, Halford S, Foster RG. The evolution of irradiance detection: melanopsin and the non-visual opsins. Philosophical transactions of the Royal Society of London Series B, Biological sciences. 2009;364(1531):2849–65. 10.1098/rstb.2009.0050 19720649 PMC2781857

[14] Fernandes AM, Fero K, Arrenberg AB, Bergeron SA, Driever W, Burgess HA. Deep brain photoreceptors control light-seeking behavior in zebrafish larvae. Current biology: CB. 2012;22(21):2042–7. 10.1016/j.cub.2012.08.016 23000151 PMC3494761

[15] Foster RG, Grace MS, Provencio I, Degrip WJ, Garcia-Fernandez JM. Identification of vertebrate deep brain photoreceptors. Neuroscience and biobehavioral reviews. 1994;18(4):541–6. . 7708367 10.1016/0149-7634(94)90009-4

[16] Bromundt V, Frey S, Odermatt J, Cajochen C. Extraocular light via the ear canal does not acutely affect human circadian physiology, alertness and psychomotor vigilance performance. Chronobiology international. 2014;31(3):343–8. 10.3109/07420528.2013.854250 . 24224577

[17] Blackshaw S, Snyder SH. Encephalopsin: a novel mammalian extraretinal opsin discretely localized in the brain. The Journal of neuroscience: the official journal of the Society for Neuroscience. 1999;19(10):3681–90. . 10234000 10.1523/JNEUROSCI.19-10-03681.1999 PMC6782724

[18] Polich J, Kok A. Cognitive and biological determinants of P300: an integrative review. Biological psychology. 1995;41(2):103–46. . 8534788 10.1016/0301-0511(95)05130-9

[19] Hartikainen KM, Ogawa KH, Knight RT. Transient interference of right hemispheric function due to automatic emotional processing. Neuropsychologia. 2000;38(12):1576–80. . 11074080 10.1016/s0028-3932(00)00072-5

[20] Hartikainen KM, Waljas M, Isoviita T, Dastidar P, Liimatainen S, Solbakk AK, et al Persistent symptoms in mild to moderate traumatic brain injury associated with executive dysfunction. Journal of clinical and experimental neuropsychology. 2010;32(7):767–74. 10.1080/13803390903521000 . 20198531

[21] Hartikainen KM, Sun L, Polvivaara M, Brause M, Lehtimaki K, Haapasalo J, et al Immediate effects of deep brain stimulation of anterior thalamic nuclei on executive functions and emotion-attention interaction in humans. Journal of clinical and experimental neuropsychology. 2014;36(5):540–50. 10.1080/13803395.2014.913554 . 24839985 PMC4066928

[22] Jurvelin H, Jokelainen J, Takala T. Transcranial bright light and symptoms of jet lag: a randomized, placebo-controlled trial. Aerosp Med Hum Perform. 2015;86(4):344–50. 10.3357/AMHP.4139.2015 . 25945550

[23] Vuilleumier P, Schwartz S. Beware and be aware: capture of spatial attention by fear-related stimuli in neglect. Neuroreport. 2001;12(6):1119–22. . 11338176 10.1097/00001756-200105080-00014

[24] Vuilleumier P, Armony JL, Driver J, Dolan RJ. Distinct spatial frequency sensitivities for processing faces and emotional expressions. Nature neuroscience. 2003;6(6):624–31. 10.1038/nn1057 . 12740580

[25] Lawrence MA. ez: Easy analysis and visualization of factorial experiments. R package version 4.2–2. 2013.

[26] Kok E, Haikonen S, Luoto T, Huhtala H, Goebeler S, Haapasalo H, et al Apolipoprotein E-dependent accumulation of Alzheimer disease-related lesions begins in middle age. Annals of neurology. 2009;65(6):650–7. 10.1002/ana.21696 . 19557866

[27] Isotalo K, Kok EH, Luoto TM, Haikonen S, Haapasalo H, Lehtimaki T, et al Upstream transcription factor 1 (USF1) polymorphisms associate with Alzheimer's disease-related neuropathological lesions: Tampere Autopsy Study. Brain Pathol. 2012;22(6):765–75. 10.1111/j.1750-3639.2012.00586.x . 22390463 PMC8057645

[28] Consortium CAD. A comprehensive 1000 Genomes-based genome-wide association meta-analysis of coronary artery disease. Nature genetics. 2015. 10.1038/ng.3396 PMC4589895 26343387

[29] Ilveskoski E, Perola M, Lehtimaki T, Laippala P, Savolainen V, Pajarinen J, et al Age-dependent association of apolipoprotein E genotype with coronary and aortic atherosclerosis in middle-aged men: an autopsy study. Circulation. 1999;100(6):608–13. . 10441097 10.1161/01.cir.100.6.608

[30] Flyktman A, Manttari S, Nissila J, Timonen M, Saarela S. Transcranial light affects plasma monoamine levels and expression of brain encephalopsin in the mouse. The Journal of experimental biology. 2015;218(Pt 10):1521–6. 10.1242/jeb.111864 . 25805701

[31] Koyanagi M, Takada E, Nagata T, Tsukamoto H, Terakita A. Homologs of vertebrate Opn3 potentially serve as a light sensor in nonphotoreceptive tissue. Proceedings of the National Academy of Sciences of the United States of America. 2013;110(13):4998–5003. 10.1073/pnas.1219416110 23479626 PMC3612648

[32] Chung H, Dai T, Sharma SK, Huang YY, Carroll JD, Hamblin MR. The nuts and bolts of low-level laser (light) therapy. Ann Biomed Eng. 2012;40(2):516–33. 10.1007/s10439-011-0454-7 22045511 PMC3288797

[33] Chen AC, Arany PR, Huang YY, Tomkinson EM, Sharma SK, Kharkwal GB, et al Low-level laser therapy activates NF-kB via generation of reactive oxygen species in mouse embryonic fibroblasts. PLoS One. 2011;6(7):e22453 10.1371/journal.pone.0022453 21814580 PMC3141042

[34] Tedford CE, DeLapp S, Jacques S, Anders J. Quantitative analysis of transcranial and intraparenchymal light penetration in human cadaver brain tissue. Lasers Surg Med. 2015;47(4):312–22. 10.1002/lsm.22343 . 25772014

[35] Ferrari M, Quaresima V. A brief review on the history of human functional near-infrared spectroscopy (fNIRS) development and fields of application. NeuroImage. 2012;63(2):921–35. 10.1016/j.neuroimage.2012.03.049 . 22510258

[36] Barrett DW, Gonzalez-Lima F. Transcranial infrared laser stimulation produces beneficial cognitive and emotional effects in humans. Neuroscience. 2013;230:13–23. 10.1016/j.neuroscience.2012.11.016 . 23200785

[37] Naeser MA, Zafonte R, Krengel MH, Martin PI, Frazier J, Hamblin MR, et al Significant improvements in cognitive performance post-transcranial, red/near-infrared light-emitting diode treatments in chronic, mild traumatic brain injury: open-protocol study. J Neurotrauma. 2014;31(11):1008–17. 10.1089/neu.2013.3244 24568233 PMC4043367

[38] Luck. SJ. An Introduction to the Event-Related Potential Technique. Cambridge, Massachusetts: MIT Press, Cambridge, Massachusetts, London, England; 2005.

[39] Boly M, Garrido MI, Gosseries O, Bruno MA, Boveroux P, Schnakers C, et al Preserved feedforward but impaired top-down processes in the vegetative state. Science. 2011;332(6031):858–62. 10.1126/science.1202043 . 21566197

[40] Kouider S, Stahlhut C, Gelskov SV, Barbosa LS, Dutat M, de Gardelle V, et al A neural marker of perceptual consciousness in infants. Science. 2013;340(6130):376–80. 10.1126/science.1232509 . 23599498

[41] Hartikainen KM, Ogawa KH, Soltani M, Knight RT. Emotionally arousing stimuli compete for attention with left hemispace. Neuroreport. 2007;18(18):1929–33. 10.1097/WNR.0b013e3282f1ca18 . 18007189

[42] Hansenne M, Olin C, Pinto E, Pitchot W, Ansseau M. Event-related potentials to emotional and neutral stimuli in alcoholism. Neuropsychobiology. 2003;48(2):77–81. doi: 72881. . 14504415 10.1159/000072881

[43] Asaumi Y, Morita K, Nakashima Y, Muraoka A, Uchimura N. Evaluation of P300 components for emotion-loaded visual event-related potential in elderly subjects, including those with dementia. Psychiatry and clinical neurosciences. 2014;68(7):558–67. 10.1111/pcn.12162 . 24447302

[44] Hartikainen KM, Ogawa KH, Knight RT. Trees over forest: unpleasant stimuli compete for attention with global features. Neuroreport. 2010;21(5):344–8. 10.1097/WNR.0b013e328336eeb3 20168261 PMC2922681

[45] Polich J. Updating P300: an integrative theory of P3a and P3b. Clinical neurophysiology: official journal of the International Federation of Clinical Neurophysiology. 2007;118(10):2128–48. 10.1016/j.clinph.2007.04.019 17573239 PMC2715154

[46] Ohman A, Flykt A, Esteves F. Emotion drives attention: detecting the snake in the grass. Journal of experimental psychology General. 2001;130(3):466–78. . 11561921 10.1037/0096-3445.130.3.466

[47] Jurvelin H, Takala T, Nissila J, Timonen M, Ruger M, Jokelainen J, et al Transcranial bright light treatment via the ear canals in seasonal affective disorder: a randomized, double-blind dose-response study. BMC psychiatry. 2014;14:288 10.1186/s12888-014-0288-6 25330838 PMC4207317

