# Supplementary material for: Human Brain Reacts to Transcranial Extraocular Light
Source: PLoS One. 2016 Feb 24;11(2):e0149525. doi: 10.1371/journal.pone.0149525 (PMC4767140; doi:10.1371/journal.pone.0149525)
Supplement: S1 Table — (DOCX) [file pone.0149525.s001.docx]

**S1 Table. List of p values for the analysis of ERP time windows.** The p values of main effect and interaction effect for Emotion and Extraocular light (Extraocular light) are listed. The p values of interactions between Emotion, Extraocular light and Response type are also listed.

| Time windows (ms) | Emotion | | Extraocular light | Emotion × Extraocular light | | Emotion × Extraocular light  × Response type |
| --- | --- | --- | --- | --- | --- | --- |
| 300-400 | 0.17 | 0.40 | | | 0.26 | 0.41 |
| 400-500 | 0.02 | 0.13 | | | 0.18 | 0.80 |
| 500-600 | 0.001* | 0.12 | | | 0.62 | 0.68 |
| 600-700 | 0.08 | 0.38 | | | 0.002* | 0.86 |
| 700-800 | 0.01 | 0.50 | | | 0.002* | 0.36 |
| 800-900 | 0.10 | 0.09 | | | 0.27 | 0.43 |
| 900-1000 | 0.80 | 0.06 | | | 0.57 | 0.97 |

* being significant with the Bonferroni-adjusted p-value 0.006.
