# Supplementary material for: Human Brain Reacts to Transcranial Extraocular Light
Source: PLoS One. 2016 Feb 24;11(2):e0149525. doi: 10.1371/journal.pone.0149525 (PMC4767140; doi:10.1371/journal.pone.0149525)
Supplement: S2 Table — (DOCX) [file pone.0149525.s002.docx]

**S2 Table. Logistic regression analysis of error types did not reveal significant predictors.**

|  |  | 95% CI | |  |
| --- | --- | --- | --- | --- |
|  | OR | Lower | Upper | p value |
| Incorrect button press: | | | | |
| Emotion | 1.25 | 0.65 | 2.43 | 0.50 |
| Extraocular light | 0.96 | 0.50 | 1.78 | 0.89 |
| Emotion × Extraocular light | 1.12 | 0.44 | 2.87 | 0.81 |
| Miss errors: | | | | |
| Emotion | 0.83 | 0.25 | 2.75 | 0.76 |
| Extraocular light | 1.64 | 0.38 | 6.93 | 0.50 |
| Emotion × Extraocular light | 0.90 | 0.13 | 6.16 | 0.91 |
| Commission errors: | | | | |
| Emotion | 1.59 | 0.91 | 2.78 | 0.10 |
| Extraocular light | 1.70 | 0.96 | 3.02 | 0.07 |
| Emotion × Extraocular light | 0.63 | 0.27 | 1.47 | 0.28 |

OR = Odds Ratio; CI = 95% Confident Interval.
