# Supplementary material for: Human Brain Reacts to Transcranial Extraocular Light
Source: PLoS One. 2016 Feb 24;11(2):e0149525. doi: 10.1371/journal.pone.0149525 (PMC4767140; doi:10.1371/journal.pone.0149525)
Supplement: S1 Text — (DOCX) [file pone.0149525.s003.docx]

**S1 Text. Analysis of Event-related potential data.**

**Event-related potential time window analysis.** ERP window analysis was performed for ERPs between 300 ms and 1000ms (see Table S1). For ERP time window 500-600ms, there was a main effect of Emotion. Emotional distractors resulted in a significant ERP Difference wave (emotional – neutral) negativity (F (1, 17) = 15.46, p = 0.001; neutral 0.43 ± 2.28 µV and emotional 0.25 ± 2.27 µV); however, delivery of extraocular light did not lead to statistical difference.

There was a significant interaction effect between Emotion and Extraocular light at time window 600-700ms (F (1, 17) = 13.22, p = 0.002). Post hoc analysis revealed the main effect of Emotion only when extraocular light was OFF (F (1, 17) = 10.45, p < 0.005; neutral 3.63 ± 3.00 µV and emotional 3.33 ± 2.89 µV), but not when it was ON (F (1, 17) = 0.81, p = 0.38). Post hoc analysis of the interaction effect at time window 700-800ms (F (1, 17) = 12.86, p = 0.002) also revealed the main effect of Emotion only when extraocular light was OFF (F (1, 17) = 17.08, p = 0.0007; neutral 2.80 ± 2.14 µV and emotional 2.42 ± 1.99 µV), but not when it was ON (F (1, 17) = 0.01, p = 0.93).

**Analysis of P300 amplitude.** Since there was an interaction effect between Extraocular light and Emotion (F(1, 17) = 25.48, p = 0.0001) in the analysis of P300 amplitude, we also performed separate analysis by Emotion. When there were only emotional distractors, no main effect of Extraocular light on P300 amplitude was found (F(1, 17) = 2.07, p = 0.17). When there were only neutral distractors, no main effect of Extraocular light was found (F(1, 17) = 3.37, p = 0.08).
