# Supplementary material for: Human Brain Reacts to Transcranial Extraocular Light
Source: PLoS One. 2016 Feb 24;11(2):e0149525. doi: 10.1371/journal.pone.0149525 (PMC4767140; doi:10.1371/journal.pone.0149525)
Supplement: S2 Text — (DOCX) [file pone.0149525.s004.docx]

**S2 Text. Analysis of reaction times**

Repeated measure ANOVA analysis for reaction time found no significance for Emotion (F (1, 17) = 1.72, p = 0.21), Extraocular light (F (1, 17) = 3.33, p = 0.09), or interaction effects between Emotion and Extraocular light (F (1, 17) = 1.97, p = 0.29).
